# Supplementary material for: Synergistic Cytotoxicity of Histone Deacetylase and Poly-ADP Ribose Polymerase Inhibitors and Decitabine in Breast and Ovarian Cancer Cells: Implications for Novel Therapeutic Combinations
Source: Int J Mol Sci. 2024 Aug 26;25(17):9241. doi: 10.3390/ijms25179241 (PMC11394699; doi:10.3390/ijms25179241)
Supplement: Supplementary file 1 [file ijms-25-09241-s001.zip › ijms-3141744-supplementary.pdf]

Supplementary Table 1. Dose response models in cell proliferation. Model-adjusted differences (Contrast) the comparison between two doses each, listed in the curves of Figure 1, Estimate of the contrast, Standard Error (SE) with 95% confidence intervals (CI) and Tukey-adjusted p-values. Separate tables for breast (A) and ovarian (B) cell lines.

A. Breast cell lines

Drug	CellLine	Contrast	Estimate	SE	CI95Min	CI95Max	TukeyPValue
Panobinostat	MDAMB231	Dose10 - Dose5	-25.3	2.1	-29.3	-21.3	<.0001
Panobinostat	MDAMB231	Dose15 - Dose5	-46.1	2.1	-50.1	-42.0	<.0001
Panobinostat	MDAMB231	Dose15 - Dose10	-20.8	2.1	-24.8	-16.7	<.0001
Panobinostat	MDAMB231	Dose20 - Dose5	-58.7	2.1	-62.7	-54.7	<.0001
Panobinostat	MDAMB231	Dose20 - Dose10	-33.4	2.1	-37.4	-29.4	<.0001
Panobinostat	MDAMB231	Dose20 - Dose15	-12.6	2.1	-16.7	-8.6	<.0001
Panobinostat	MDAMB231	Dose25 - Dose5	-64.1	2.1	-68.1	-60.1	<.0001
Panobinostat	MDAMB231	Dose25 - Dose10	-38.8	2.1	-42.8	-34.8	<.0001
Panobinostat	MDAMB231	Dose25 - Dose15	-18.0	2.1	-22.1	-14.0	<.0001
Panobinostat	MDAMB231	Dose25 - Dose20	-5.4	2.1	-9.4	-1.4	0.11
Panobinostat	MDAMB231	Dose30 - Dose5	-69.0	2.1	-73.0	-65.0	<.0001
Panobinostat	MDAMB231	Dose30 - Dose10	-43.7	2.1	-47.7	-39.7	<.0001
Panobinostat	MDAMB231	Dose30 - Dose15	-22.9	2.1	-27.0	-18.9	<.0001
Panobinostat	MDAMB231	Dose30 - Dose20	-10.3	2.1	-14.3	-6.3	0.0001

Panobinostat	MDAMB231	Dose30 - Dose25	-4.9	2.1	-8.9	-0.9	0.18
Panobinostat	MCF7	Dose10 - Dose5	-11.7	2.3	-16.2	-7.2	<.0001
Panobinostat	MCF7	Dose15 - Dose5	-22.5	2.3	-27.0	-18.0	<.0001
Panobinostat	MCF7	Dose15 - Dose10	-10.8	2.3	-15.3	-6.3	0.0003
Panobinostat	MCF7	Dose20 - Dose5	-34.5	2.3	-38.9	-30.0	<.0001
Panobinostat	MCF7	Dose20 - Dose10	-22.8	2.3	-27.3	-18.3	<.0001
Panobinostat	MCF7	Dose20 - Dose15	-12.0	2.3	-16.4	-7.5	<.0001
Panobinostat	MCF7	Dose25 - Dose5	-40.9	2.3	-45.3	-36.4	<.0001
Panobinostat	MCF7	Dose25 - Dose10	-29.2	2.3	-33.6	-24.7	<.0001
Panobinostat	MCF7	Dose25 - Dose15	-18.3	2.3	-22.8	-13.9	<.0001
Panobinostat	MCF7	Dose25 - Dose20	-6.4	2.3	-10.9	-1.9	0.07
Panobinostat	MCF7	Dose30 - Dose5	-51.0	2.3	-55.5	-46.5	<.0001
Panobinostat	MCF7	Dose30 - Dose10	-39.3	2.3	-43.8	-34.8	<.0001
Panobinostat	MCF7	Dose30 - Dose15	-28.5	2.3	-33.0	-24.0	<.0001
Panobinostat	MCF7	Dose30 - Dose20	-16.5	2.3	-21.0	-12.0	<.0001
Panobinostat	MCF7	Dose30 - Dose25	-10.1	2.3	-14.6	-5.6	0.0007
Vorinostat_SAHA	MDAMB231	Dose1 - Dose0.5	-4.0	3.0	-9.9	2.0	0.78
Vorinostat_SAHA	MDAMB231	Dose1.5 - Dose0.5	-15.0	3.0	-21.0	-9.1	0.0002
Vorinostat_SAHA	MDAMB231	Dose1.5 - Dose1	-11.1	2.4	-15.9	-6.3	0.0006
Vorinostat_SAHA	MDAMB231	Dose2 - Dose0.5	-37.0	3.0	-42.9	-31.0	<.0001

Vorinostat_SAHA	MDAMB231	Dose2 - Dose1	-33.0	2.4	-37.8	-28.2	<.0001
Vorinostat_SAHA	MDAMB231	Dose2 - Dose1.5	-21.9	2.4	-26.7	-17.1	<.0001
Vorinostat_SAHA	MDAMB231	Dose2.5 - Dose0.5	-49.3	3.0	-55.2	-43.3	<.0001
Vorinostat_SAHA	MDAMB231	Dose2.5 - Dose1	-45.3	2.4	-50.1	-40.5	<.0001
Vorinostat_SAHA	MDAMB231	Dose2.5 - Dose1.5	-34.3	2.4	-39.1	-29.5	<.0001
Vorinostat_SAHA	MDAMB231	Dose2.5 - Dose2	-12.3	2.4	-17.1	-7.5	0.0001
Vorinostat_SAHA	MDAMB231	Dose3 - Dose0.5	-63.4	3.0	-69.3	-57.4	<.0001
Vorinostat_SAHA	MDAMB231	Dose3 - Dose1	-59.4	2.4	-64.2	-54.6	<.0001
Vorinostat_SAHA	MDAMB231	Dose3 - Dose1.5	-48.3	2.4	-53.1	-43.5	<.0001
Vorinostat_SAHA	MDAMB231	Dose3 - Dose2	-26.4	2.4	-31.2	-21.6	<.0001
Vorinostat_SAHA	MDAMB231	Dose3 - Dose2.5	-14.1	2.4	-18.9	-9.3	<.0001
Vorinostat_SAHA	MCF7	Dose1 - Dose0.5	-7.4	3.0	-13.2	-1.6	0.14
Vorinostat_SAHA	MCF7	Dose1.5 - Dose0.5	-16.7	2.9	-22.3	-11.0	<.0001
Vorinostat_SAHA	MCF7	Dose1.5 - Dose1	-9.3	2.9	-14.9	-3.6	0.026
Vorinostat_SAHA	MCF7	Dose2 - Dose0.5	-28.1	2.9	-33.7	-22.4	<.0001
Vorinostat_SAHA	MCF7	Dose2 - Dose1	-20.7	2.9	-26.3	-15.0	<.0001
Vorinostat_SAHA	MCF7	Dose2 - Dose1.5	-11.4	2.8	-16.9	-5.9	0.002
Vorinostat_SAHA	MCF7	Dose2.5 - Dose0.5	-32.8	2.9	-38.5	-27.2	<.0001

Vorinostat_SAHA	MCF7	Dose2.5 - Dose1	-25.4	2.9	-31.1	-19.8	<.0001
Vorinostat_SAHA	MCF7	Dose2.5 - Dose1.5	-16.1	2.8	-21.6	-10.6	<.0001
Vorinostat_SAHA	MCF7	Dose2.5 - Dose2	-4.7	2.8	-10.2	0.8	0.54
Vorinostat_SAHA	MCF7	Dose3 - Dose0.5	-42.8	2.9	-48.4	-37.1	<.0001
Vorinostat_SAHA	MCF7	Dose3 - Dose1	-35.4	2.9	-41.0	-29.7	<.0001
Vorinostat_SAHA	MCF7	Dose3 - Dose1.5	-26.1	2.8	-31.6	-20.6	<.0001
Vorinostat_SAHA	MCF7	Dose3 - Dose2	-14.7	2.8	-20.2	-9.2	<.0001
Vorinostat_SAHA	MCF7	Dose3 - Dose2.5	-10.0	2.8	-15.4	-4.5	0.011
Talazoparib	MDAMB231	Dose0.2 - Dose0.1	-1.8	1.8	-5.3	1.7	0.91
Talazoparib	MDAMB231	Dose0.3 - Dose0.1	-5.2	1.8	-8.7	-1.7	0.056
Talazoparib	MDAMB231	Dose0.3 - Dose0.2	-3.4	1.8	-6.9	0.1	0.41
Talazoparib	MDAMB231	Dose0.4 - Dose0.1	-6.2	1.8	-9.7	-2.7	0.012
Talazoparib	MDAMB231	Dose0.4 - Dose0.2	-4.4	1.8	-7.9	-0.9	0.15
Talazoparib	MDAMB231	Dose0.4 - Dose0.3	-1.0	1.8	-4.5	2.5	0.99
Talazoparib	MDAMB231	Dose0.5 - Dose0.1	-8.9	1.8	-12.4	-5.4	0.0001

Talazoparib	MDAMB231	Dose0.5 - Dose0.2	-7.1	1.8	-10.6	-3.6	0.003
Talazoparib	MDAMB231	Dose0.5 - Dose0.3	-3.7	1.8	-7.2	-0.2	0.32
Talazoparib	MDAMB231	Dose0.5 - Dose0.4	-2.7	1.8	-6.2	0.8	0.67
Talazoparib	MDAMB231	Dose0.6 - Dose0.1	-16.4	1.8	-19.9	-12.9	<.0001
Talazoparib	MDAMB231	Dose0.6 - Dose0.2	-14.6	1.8	-18.1	-11.1	<.0001
Talazoparib	MDAMB231	Dose0.6 - Dose0.3	-11.2	1.8	-14.7	-7.7	<.0001
Talazoparib	MDAMB231	Dose0.6 - Dose0.4	-10.1	1.8	-13.6	-6.6	<.0001
Talazoparib	MDAMB231	Dose0.6 - Dose0.5	-7.5	1.8	-11.0	-4.0	0.002
Talazoparib	MCF7	Dose0.2 - Dose0.1	-3.4	1.7	-6.7	0.0	0.38
Talazoparib	MCF7	Dose0.3 - Dose0.1	-4.9	1.7	-8.2	-1.5	0.067
Talazoparib	MCF7	Dose0.3 - Dose0.2	-1.5	1.7	-4.8	1.7	0.94
Talazoparib	MCF7	Dose0.4 - Dose0.1	-7.0	1.7	-10.3	-3.6	0.002

Talazoparib	MCF7	Dose0.4 - Dose0.2	-3.6	1.7	-6.9	-0.4	0.27
Talazoparib	MCF7	Dose0.4 - Dose0.3	-2.1	1.7	-5.4	1.2	0.8
Talazoparib	MCF7	Dose0.5 - Dose0.1	-10.0	1.7	-13.3	-6.6	<.0001
Talazoparib	MCF7	Dose0.5 - Dose0.2	-6.6	1.7	-9.9	-3.4	0.003
Talazoparib	MCF7	Dose0.5 - Dose0.3	-5.1	1.7	-8.4	-1.9	0.038
Talazoparib	MCF7	Dose0.5 - Dose0.4	-3.0	1.7	-6.3	0.2	0.47
Talazoparib	MCF7	Dose0.6 - Dose0.1	-17.0	1.7	-20.4	-13.6	<.0001
Talazoparib	MCF7	Dose0.6 - Dose0.2	-13.6	1.7	-16.9	-10.4	<.0001
Talazoparib	MCF7	Dose0.6 - Dose0.3	-12.1	1.7	-15.4	-8.9	<.0001
Talazoparib	MCF7	Dose0.6 - Dose0.4	-10.0	1.7	-13.3	-6.8	<.0001
Talazoparib	MCF7	Dose0.6 - Dose0.5	-7.0	1.7	-10.3	-3.8	0.001
Olaparib	MDAMB231	Dose10 - Dose5	-5.3	1.9	-9.1	-1.6	0.08
Olaparib	MDAMB231	Dose15 - Dose5	-10.0	1.9	-13.6	-6.3	<.0001
Olaparib	MDAMB231	Dose15 - Dose10	-4.7	1.9	-8.4	-0.9	0.16

Olaparib	MDAMB231	Dose20 - Dose5	-16.8	1.9	-20.4	-13.2	<.0001
Olaparib	MDAMB231	Dose20 - Dose10	-11.5	1.9	-15.2	-7.7	<.0001
Olaparib	MDAMB231	Dose20 - Dose15	-6.8	1.9	-10.5	-3.2	0.007
Olaparib	MDAMB231	Dose25 - Dose5	-20.8	1.9	-24.5	-17.2	<.0001
Olaparib	MDAMB231	Dose25 - Dose10	-15.5	1.9	-19.3	-11.8	<.0001
Olaparib	MDAMB231	Dose25 - Dose15	-10.9	1.9	-14.5	-7.2	<.0001
Olaparib	MDAMB231	Dose25 - Dose20	-4.0	1.9	-7.7	-0.4	0.27
Olaparib	MDAMB231	Dose30 - Dose5	-27.5	1.9	-31.1	-23.8	<.0001
Olaparib	MDAMB231	Dose30 - Dose10	-22.1	1.9	-25.9	-18.4	<.0001
Olaparib	MDAMB231	Dose30 - Dose15	-17.5	1.9	-21.1	-13.8	<.0001
Olaparib	MDAMB231	Dose30 - Dose20	-10.7	1.9	-14.3	-7.0	<.0001
Olaparib	MDAMB231	Dose30 - Dose25	-6.6	1.9	-10.3	-3.0	0.01
Olaparib	MCF7	Dose10 - Dose5	-8.2	3.0	-14.1	-2.4	0.08
Olaparib	MCF7	Dose15 - Dose5	-13.5	3.0	-19.4	-7.7	0.0005
Olaparib	MCF7	Dose15 - Dose10	-5.3	3.0	-11.2	0.6	0.49
Olaparib	MCF7	Dose20 - Dose5	-20.7	3.0	-26.6	-14.9	<.0001
Olaparib	MCF7	Dose20 - Dose10	-12.5	3.0	-18.4	-6.6	0.002
Olaparib	MCF7	Dose20 - Dose15	-7.2	3.0	-13.1	-1.3	0.18
Olaparib	MCF7	Dose25 - Dose5	-25.0	3.0	-30.9	-19.2	<.0001
Olaparib	MCF7	Dose25 - Dose10	-16.8	3.0	-22.7	-10.9	<.0001

Olaparib	MCF7	Dose25 - Dose15	-11.5	3.0	-17.4	-5.6	0.004
Olaparib	MCF7	Dose25 - Dose20	-4.3	3.0	-10.2	1.6	0.7
Olaparib	MCF7	Dose30 - Dose5	-32.1	3.0	-37.9	-26.2	<.0001
Olaparib	MCF7	Dose30 - Dose10	-23.8	3.0	-29.7	-18.0	<.0001
Olaparib	MCF7	Dose30 - Dose15	-18.5	3.0	-24.4	-12.7	<.0001
Olaparib	MCF7	Dose30 - Dose20	-11.4	3.0	-17.2	-5.5	0.005
Olaparib	MCF7	Dose30 - Dose25	-7.0	3.0	-12.9	-1.2	0.19
Decitabine	MDAMB231	Dose20 - Dose10	-5.0	1.2	-7.3	-2.7	0.001
Decitabine	MDAMB231	Dose30 - Dose10	-7.8	1.2	-10.1	-5.5	<.0001
Decitabine	MDAMB231	Dose30 - Dose20	-2.8	1.2	-5.1	-0.5	0.17
Decitabine	MDAMB231	Dose40 - Dose10	-9.1	1.2	-11.4	-6.8	<.0001
Decitabine	MDAMB231	Dose40 - Dose20	-4.1	1.2	-6.4	-1.8	0.012
Decitabine	MDAMB231	Dose40 - Dose30	-1.3	1.2	-3.6	1.0	0.88
Decitabine	MDAMB231	Dose50 - Dose10	-10.1	1.2	-12.5	-7.8	<.0001
Decitabine	MDAMB231	Dose50 - Dose20	-5.2	1.2	-7.5	-2.8	0.001
Decitabine	MDAMB231	Dose50 - Dose30	-2.3	1.2	-4.7	0.0	0.39
Decitabine	MDAMB231	Dose50 - Dose40	-1.1	1.2	-3.4	1.3	0.95
Decitabine	MDAMB231	Dose60 - Dose10	-14.1	1.2	-16.4	-11.8	<.0001
Decitabine	MDAMB231	Dose60 - Dose20	-9.1	1.2	-11.4	-6.8	<.0001
Decitabine	MDAMB231	Dose60 - Dose30	-6.3	1.2	-8.6	-4.0	<.0001

Decitabine	MDAMB231	Dose60 - Dose40	-5.0	1.2	-7.3	-2.7	0.001
Decitabine	MDAMB231	Dose60 - Dose50	-4.0	1.2	-6.3	-1.6	0.022
Decitabine	MCF7	Dose20 - Dose10	-4.6	2.4	-9.4	0.1	0.41
Decitabine	MCF7	Dose30 - Dose10	-5.4	2.4	-10.1	-0.8	0.21
Decitabine	MCF7	Dose30 - Dose20	-0.8	2.4	-5.6	4.0	1
Decitabine	MCF7	Dose40 - Dose10	-9.1	2.4	-13.7	-4.4	0.005
Decitabine	MCF7	Dose40 - Dose20	-4.4	2.4	-9.2	0.3	0.46
Decitabine	MCF7	Dose40 - Dose30	-3.6	2.4	-8.2	1.0	0.64
Decitabine	MCF7	Dose50 - Dose10	-13.5	2.4	-18.2	-8.7	<.0001
Decitabine	MCF7	Dose50 - Dose20	-8.8	2.5	-13.7	-4.0	0.011
Decitabine	MCF7	Dose50 - Dose30	-8.0	2.4	-12.8	-3.3	0.021
Decitabine	MCF7	Dose50 - Dose40	-4.4	2.4	-9.2	0.3	0.46
Decitabine	MCF7	Dose60 - Dose10	-19.2	2.4	-24.0	-14.5	<.0001
Decitabine	MCF7	Dose60 - Dose20	-14.6	2.5	-19.5	-9.7	<.0001
Decitabine	MCF7	Dose60 - Dose30	-13.8	2.4	-18.5	-9.0	<.0001
Decitabine	MCF7	Dose60 - Dose40	-10.2	2.4	-14.9	-5.4	0.002
Decitabine	MCF7	Dose60 - Dose50	-5.7	2.5	-10.6	-0.9	0.21

B. Ovarian cell lines

Drug	CellLine	Contrast	Estimate	SE	CI95Min	CI95Max	TukeyPValue
Panobinostat	HEY T30	Dose20 - Dose10	-2.5	2.4	-7.3	2.2	0.9
Panobinostat	HEY T30	Dose30 - Dose10	-12.0	2.1	-16.1	-7.9	<.0001
Panobinostat	HEY T30	Dose30 - Dose20	-9.4	2.4	-14.2	-4.7	0.005
Panobinostat	HEY T30	Dose40 - Dose10	-17.4	2.1	-21.5	-13.3	<.0001
Panobinostat	HEY T30	Dose40 - Dose20	-14.9	2.4	-19.6	-10.1	<.0001
Panobinostat	HEY T30	Dose40 - Dose30	-5.4	2.1	-9.5	-1.3	0.13
Panobinostat	HEY T30	Dose50 - Dose10	-25.5	2.1	-29.7	-21.4	<.0001
Panobinostat	HEY T30	Dose50 - Dose20	-23.0	2.4	-27.8	-18.2	<.0001
Panobinostat	HEY T30	Dose50 - Dose30	-13.6	2.1	-17.7	-9.4	<.0001
Panobinostat	HEY T30	Dose50 - Dose40	-8.1	2.1	-12.2	-4.0	0.005
Panobinostat	HEY T30	Dose60 - Dose10	-31.1	2.1	-35.2	-27.0	<.0001

Panobinostat	HEY T30	Dose60 - Dose20	-28.6	2.4	-33.3	-23.8	<.0001
Panobinostat	HEY T30	Dose60 - Dose30	-19.1	2.1	-23.3	-15.0	<.0001
Panobinostat	HEY T30	Dose60 - Dose40	-13.7	2.1	-17.8	-9.6	<.0001
Panobinostat	HEY T30	Dose60 - Dose50	-5.6	2.1	-9.7	-1.5	0.11
Panobinostat	SKOV-3	Dose20 - Dose10	-11.4	2.2	-15.6	-7.1	<.0001
Panobinostat	SKOV-3	Dose30 - Dose10	-17.2	2.2	-21.4	-12.9	<.0001
Panobinostat	SKOV-3	Dose30 - Dose20	-5.8	2.2	-10.0	-1.5	0.11
Panobinostat	SKOV-3	Dose40 - Dose10	-20.3	2.2	-24.5	-16.0	<.0001
Panobinostat	SKOV-3	Dose40 - Dose20	-8.9	2.2	-13.2	-4.6	0.003
Panobinostat	SKOV-3	Dose40 - Dose30	-3.1	2.2	-7.4	1.2	0.71
Panobinostat	SKOV-3	Dose50 - Dose10	-22.0	2.2	-26.2	-17.7	<.0001
Panobinostat	SKOV-3	Dose50 - Dose20	-10.6	2.2	-14.8	-6.3	0.0003

Panobinostat	SKOV-3	Dose50 - Dose30	-4.8	2.2	-9.1	-0.5	0.26
Panobinostat	SKOV-3	Dose50 - Dose40	-1.7	2.2	-6.0	2.6	0.97
Panobinostat	SKOV-3	Dose60 - Dose10	-25.8	2.2	-30.0	-21.5	<.0001
Panobinostat	SKOV-3	Dose60 - Dose20	-14.4	2.2	-18.7	-10.1	<.0001
Panobinostat	SKOV-3	Dose60 - Dose30	-8.6	2.2	-12.9	-4.3	0.004
Panobinostat	SKOV-3	Dose60 - Dose40	-5.5	2.2	-9.8	-1.2	0.14
Panobinostat	SKOV-3	Dose60 - Dose50	-3.8	2.2	-8.1	0.5	0.51
Vorinostat_SAHA	HEY T30	Dose2 - Dose1	-8.9	1.3	-11.4	-6.3	<.0001
Vorinostat_SAHA	HEY T30	Dose3 - Dose1	-14.5	1.3	-17.0	-11.9	<.0001
Vorinostat_SAHA	HEY T30	Dose3 - Dose2	-5.6	1.3	-8.2	-3.1	0.001
Vorinostat_SAHA	HEY T30	Dose4 - Dose1	-18.9	1.3	-21.4	-16.3	<.0001
Vorinostat_SAHA	HEY T30	Dose4 - Dose2	-10.0	1.3	-12.5	-7.4	<.0001

Vorinostat_SAHA	HEY T30	Dose4 - Dose3	-4.4	1.3	-6.9	-1.8	0.02
Vorinostat_SAHA	HEY T30	Dose5 - Dose1	-24.6	1.3	-27.2	-22.1	<.0001
Vorinostat_SAHA	HEY T30	Dose5 - Dose2	-15.8	1.3	-18.3	-13.2	<.0001
Vorinostat_SAHA	HEY T30	Dose5 - Dose3	-10.1	1.3	-12.7	-7.6	<.0001
Vorinostat_SAHA	HEY T30	Dose5 - Dose4	-5.8	1.3	-8.3	-3.2	0.001
Vorinostat_SAHA	HEY T30	Dose6 - Dose1	-28.4	1.3	-30.9	-25.8	<.0001
Vorinostat_SAHA	HEY T30	Dose6 - Dose2	-19.5	1.3	-22.1	-16.9	<.0001
Vorinostat_SAHA	HEY T30	Dose6 - Dose3	-13.9	1.3	-16.4	-11.3	<.0001
Vorinostat_SAHA	HEY T30	Dose6 - Dose4	-9.5	1.3	-12.1	-7.0	<.0001
Vorinostat_SAHA	HEY T30	Dose6 - Dose5	-3.7	1.3	-6.3	-1.2	0.066
Vorinostat_SAHA	SKOV-3	Dose2 - Dose1	-13.1	2.3	-17.6	-8.6	<.0001
Vorinostat_SAHA	SKOV-3	Dose3 - Dose1	-20.4	2.3	-24.9	-15.9	<.0001
Vorinostat_SAHA	SKOV-3	Dose3 - Dose2	-7.3	2.3	-11.8	-2.8	0.033
Vorinostat_SAHA	SKOV-3	Dose4 - Dose1	-26.9	2.3	-31.4	-22.3	<.0001

Vorinostat_SAHA	SKOV-3	Dose4 - Dose2	-13.7	2.3	-18.3	-9.2	<.0001
Vorinostat_SAHA	SKOV-3	Dose4 - Dose3	-6.5	2.3	-11.0	-1.9	0.08
Vorinostat_SAHA	SKOV-3	Dose5 - Dose1	-29.1	2.3	-33.7	-24.6	<.0001
Vorinostat_SAHA	SKOV-3	Dose5 - Dose2	-16.0	2.3	-20.5	-11.5	<.0001
Vorinostat_SAHA	SKOV-3	Dose5 - Dose3	-8.7	2.3	-13.2	-4.2	0.006
Vorinostat_SAHA	SKOV-3	Dose5 - Dose4	-2.3	2.3	-6.8	2.2	0.92
Vorinostat_SAHA	SKOV-3	Dose6 - Dose1	-33.6	2.3	-38.1	-29.1	<.0001
Vorinostat_SAHA	SKOV-3	Dose6 - Dose2	-20.5	2.3	-25.0	-15.9	<.0001
Vorinostat_SAHA	SKOV-3	Dose6 - Dose3	-13.2	2.3	-17.7	-8.7	<.0001
Vorinostat_SAHA	SKOV-3	Dose6 - Dose4	-6.7	2.3	-11.2	-2.2	0.06
Vorinostat_SAHA	SKOV-3	Dose6 - Dose5	-4.4	2.3	-9.0	0.1	0.4
Talazoparib	HEY T30	Dose2 - Dose1	-3.8	3.3	-10.2	2.6	0.85
Talazoparib	HEY T30	Dose3 - Dose1	-6.6	3.3	-12.9	-0.2	0.35
Talazoparib	HEY T30	Dose3 - Dose2	-2.8	3.3	-9.1	3.6	0.96
Talazoparib	HEY T30	Dose4 - Dose1	-8.3	3.9	-16.0	-0.6	0.3
Talazoparib	HEY T30	Dose4 - Dose2	-4.5	3.9	-12.2	3.2	0.86
Talazoparib	HEY T30	Dose4 - Dose3	-1.7	3.9	-9.4	6.0	1

Talazoparib	HEY T30	Dose5 - Dose1	-12.6	3.3	-19.0	-6.2	0.005
Talazoparib	HEY T30	Dose5 - Dose2	-8.8	3.3	-15.2	-2.4	0.1
Talazoparib	HEY T30	Dose5 - Dose3	-6.0	3.3	-12.4	0.4	0.45
Talazoparib	HEY T30	Dose5 - Dose4	-4.3	3.9	-12.0	3.4	0.88
Talazoparib	HEY T30	Dose6 - Dose1	-23.5	3.3	-29.8	-17.1	<.0001
Talazoparib	HEY T30	Dose6 - Dose2	-19.7	3.3	-26.0	-13.3	<.0001
Talazoparib	HEY T30	Dose6 - Dose3	-16.9	3.3	-23.3	-10.5	<.0001
Talazoparib	HEY T30	Dose6 - Dose4	-15.2	3.9	-22.9	-7.5	0.005
Talazoparib	HEY T30	Dose6 - Dose5	-10.9	3.3	-17.3	-4.5	0.02
Talazoparib	SKOV-3	Dose2 - Dose1	-7.8	1.9	-11.6	-4.1	0.006
Talazoparib	SKOV-3	Dose3 - Dose1	-9.8	1.9	-13.6	-6.0	0.0005
Talazoparib	SKOV-3	Dose3 - Dose2	-1.9	1.9	-5.7	1.8	0.91
Talazoparib	SKOV-3	Dose4 - Dose1	-10.7	1.9	-14.4	-6.9	0.0002
Talazoparib	SKOV-3	Dose4 - Dose2	-2.8	1.9	-6.6	0.9	0.69
Talazoparib	SKOV-3	Dose4 - Dose3	-0.9	1.9	-4.7	2.9	1

Talazoparib	SKOV-3	Dose5 - Dose1	-11.5	2.0	-15.5	-7.5	0.0002
Talazoparib	SKOV-3	Dose5 - Dose2	-3.6	2.0	-7.6	0.4	0.5
Talazoparib	SKOV-3	Dose5 - Dose3	-1.7	2.0	-5.7	2.3	0.96
Talazoparib	SKOV-3	Dose5 - Dose4	-0.8	2.0	-4.8	3.2	1
Talazoparib	SKOV-3	Dose6 - Dose1	-13.8	1.9	-17.5	-10.0	<.0001
Talazoparib	SKOV-3	Dose6 - Dose2	-5.9	1.9	-9.7	-2.2	0.054
Talazoparib	SKOV-3	Dose6 - Dose3	-4.0	1.9	-7.8	-0.2	0.34
Talazoparib	SKOV-3	Dose6 - Dose4	-3.1	1.9	-6.9	0.7	0.6
Talazoparib	SKOV-3	Dose6 - Dose5	-2.3	2.0	-6.3	1.7	0.87
Olaparib	HEY T30	Dose20 - Dose10	-10.6	2.1	-14.8	-6.4	0.0002
Olaparib	HEY T30	Dose30 - Dose10	-16.4	1.9	-20.2	-12.6	<.0001
Olaparib	HEY T30	Dose30 - Dose20	-5.8	2.1	-10.0	-1.6	0.1
Olaparib	HEY T30	Dose40 - Dose10	-20.1	2.0	-24.0	-16.1	<.0001
Olaparib	HEY T30	Dose40 - Dose20	-9.5	2.2	-13.7	-5.2	0.001
Olaparib	HEY T30	Dose40 - Dose30	-3.7	2.0	-7.6	0.3	0.47
Olaparib	HEY T30	Dose50 - Dose10	-20.6	2.0	-24.5	-16.6	<.0001

Olaparib	HEY T30	Dose50 - Dose20	-10.0	2.2	-14.2	-5.7	0.0008
Olaparib	HEY T30	Dose50 - Dose30	-4.2	2.0	-8.1	-0.2	0.33
Olaparib	HEY T30	Dose50 - Dose40	-0.5	2.1	-4.6	3.6	1
Olaparib	HEY T30	Dose60 - Dose10	-23.0	1.9	-26.8	-19.1	<.0001
Olaparib	HEY T30	Dose60 - Dose20	-12.4	2.1	-16.6	-8.2	<.0001
Olaparib	HEY T30	Dose60 - Dose30	-6.6	1.9	-10.4	-2.8	0.021
Olaparib	HEY T30	Dose60 - Dose40	-2.9	2.0	-6.9	1.1	0.71
Olaparib	HEY T30	Dose60 - Dose50	-2.4	2.0	-6.4	1.6	0.84
Olaparib	SKOV-3	Dose20 - Dose10	-3.1	2.6	-8.1	1.9	0.83
Olaparib	SKOV-3	Dose30 - Dose10	-5.1	2.6	-10.1	-0.1	0.38
Olaparib	SKOV-3	Dose30 - Dose20	-2.0	2.6	-7.0	3.0	0.97
Olaparib	SKOV-3	Dose40 - Dose10	-7.9	3.2	-14.2	-1.6	0.19

Olaparib	SKOV-3	Dose40 - Dose20	-4.8	3.2	-11.1	1.5	0.68
Olaparib	SKOV-3	Dose40 - Dose30	-2.8	3.2	-9.1	3.6	0.95
Olaparib	SKOV-3	Dose50 - Dose10	-11.2	3.2	-17.5	-4.9	0.024
Olaparib	SKOV-3	Dose50 - Dose20	-8.1	3.2	-14.4	-1.8	0.16
Olaparib	SKOV-3	Dose50 - Dose30	-6.1	3.2	-12.4	0.2	0.43
Olaparib	SKOV-3	Dose50 - Dose40	-3.4	3.6	-10.5	3.7	0.93
Olaparib	SKOV-3	Dose60 - Dose10	-15.0	2.9	-20.6	-9.3	0.0005
Olaparib	SKOV-3	Dose60 - Dose20	-11.9	2.9	-17.5	-6.2	0.006
Olaparib	SKOV-3	Dose60 - Dose30	-9.9	2.9	-15.5	-4.2	0.027
Olaparib	SKOV-3	Dose60 - Dose40	-7.1	3.4	-13.8	-0.4	0.34
Olaparib	SKOV-3	Dose60 - Dose50	-3.7	3.4	-10.4	3.0	0.88
Decitabine	HEY T30	Dose4 - Dose2	-4.3	2.0	-8.3	-0.3	0.3

Decitabine	HEY T30	Dose6 - Dose2	-4.7	1.9	-8.4	-1.0	0.15
Decitabine	HEY T30	Dose6 - Dose4	-0.4	2.0	-4.4	3.6	1
Decitabine	HEY T30	Dose8 - Dose2	-5.7	1.8	-9.3	-2.1	0.04
Decitabine	HEY T30	Dose8 - Dose4	-1.3	2.0	-5.2	2.6	0.98
Decitabine	HEY T30	Dose8 - Dose6	-1.0	1.8	-4.5	2.6	1
Decitabine	HEY T30	Dose10 - Dose2	-7.7	1.9	-11.4	-4.0	0.003
Decitabine	HEY T30	Dose10 - Dose4	-3.4	2.1	-7.4	0.7	0.58
Decitabine	HEY T30	Dose10 - Dose6	-3.0	1.9	-6.7	0.7	0.61
Decitabine	HEY T30	Dose10 - Dose8	-2.0	1.8	-5.6	1.6	0.87
Decitabine	HEY T30	Dose12 - Dose2	-13.1	1.8	-16.7	-9.5	<.0001
Decitabine	HEY T30	Dose12 - Dose4	-8.8	2.0	-12.7	-4.9	0.001
Decitabine	HEY T30	Dose12 - Dose6	-8.4	1.8	-12.0	-4.8	0.0006

Decitabine	HEY T30	Dose12 - Dose8	-7.5	1.8	-10.9	-4.0	0.002
Decitabine	HEY T30	Dose12 - Dose10	-5.4	1.8	-9.0	-1.8	0.055
Decitabine	SKOV-3	Dose4 - Dose2	-4.8	1.6	-7.9	-1.6	0.07
Decitabine	SKOV-3	Dose6 - Dose2	-7.3	1.6	-10.4	-4.1	0.003
Decitabine	SKOV-3	Dose6 - Dose4	-2.5	1.6	-5.6	0.7	0.64
Decitabine	SKOV-3	Dose8 - Dose2	-7.8	1.7	-11.2	-4.4	0.003
Decitabine	SKOV-3	Dose8 - Dose4	-3.0	1.7	-6.4	0.4	0.52
Decitabine	SKOV-3	Dose8 - Dose6	-0.5	1.7	-3.9	2.8	1
Decitabine	SKOV-3	Dose10 - Dose2	-8.9	1.9	-12.6	-5.1	0.002
Decitabine	SKOV-3	Dose10 - Dose4	-4.1	1.9	-7.8	-0.3	0.31
Decitabine	SKOV-3	Dose10 - Dose6	-1.6	1.9	-5.4	2.1	0.96
Decitabine	SKOV-3	Dose10 - Dose8	-1.1	2.0	-4.9	2.8	0.99
Decitabine	SKOV-3	Dose12 - Dose2	-17.4	1.9	-21.2	-13.7	<.0001
Decitabine	SKOV-3	Dose12 - Dose4	-12.7	1.9	-16.4	-8.9	<.0001
Decitabine	SKOV-3	Dose12 - Dose6	-10.2	1.9	-13.9	-6.4	0.0006
Decitabine	SKOV-3	Dose12 - Dose8	-9.6	2.0	-13.5	-5.8	0.001
Decitabine	SKOV-3	Dose12 - Dose10	-8.6	2.1	-12.6	-4.5	0.007

Supplementary Table 2. Colony formation model-adjusted differences (Contrasts) between drug combinations and their component drugs, listed in Figure 3A, Estimate of the contrast, Standard Error (SE) with 95% confidence intervals (CI) and Hommel-adjusted p-values. Separate tables for breast (A) and ovarian (B) cultures.

A. Breast cell lines

CellLine	Contrast	Estimate	SE	CI95Min	CI95Max	HommelPValue
MDAMB231	PT - Pano	-42.3	18.2	-78.0	-6.5	1
MDAMB231	PT - TLZ	-9.2	18.2	-44.9	26.6	1
MDAMB231	PO - Pano	-34.1	18.2	-69.8	1.7	1
MDAMB231	PO - Ola	-16.5	18.2	-52.2	19.3	1
MDAMB231	ST - SAHA	-53.5	18.2	-89.3	-17.8	1
MDAMB231	ST - TLZ	-12.6	18.2	-48.3	23.2	1
MDAMB231	SO - SAHA	-39.7	18.2	-75.4	-3.9	1
MDAMB231	SO - Ola	-14.2	18.2	-50.0	21.5	1
MDAMB231	PTD - Pano	-69.0	18.2	-104.8	-33.3	1
MDAMB231	PTD - TLZ	-35.9	18.2	-71.7	-0.2	1
MDAMB231	PTD - DAC	-61.8	18.2	-97.5	-26.0	1
MDAMB231	POD - Pano	-53.0	18.2	-88.7	-17.2	1
MDAMB231	POD - Ola	-35.4	18.2	-71.2	0.3	1
MDAMB231	POD - DAC	-45.7	18.2	-81.5	-10.0	1

MDAMB231	STD - SAHA	-78.9	18.2	-114.6	-43.1	0.54
MDAMB231	STD - TLZ	-37.9	18.2	-73.7	-2.2	1
MDAMB231	STD - DAC	-63.7	18.2	-99.5	-28.0	1
MDAMB231	SOD - SAHA	-67.4	18.2	-103.2	-31.7	1
MDAMB231	SOD - Ola	-42.0	18.2	-77.7	-6.2	1
MDAMB231	SOD - DAC	-52.3	18.2	-88.0	-16.5	1
MCF7	PT - Pano	-21.6	10.4	-42.0	-1.2	1
MCF7	PT - TLZ	-6.2	10.4	-26.6	14.1	1
MCF7	PO - Pano	-30.2	10.4	-50.5	-9.8	1
MCF7	PO - Ola	-15.4	10.4	-35.8	5.0	1
MCF7	ST - SAHA	-25.2	10.4	-45.5	-4.8	1
MCF7	ST - TLZ	-3.3	10.4	-23.6	17.1	1
MCF7	SO - SAHA	-31.2	10.4	-51.6	-10.8	1
MCF7	SO - Ola	-9.9	10.4	-30.2	10.5	1
MCF7	PTD - Pano	-30.2	10.4	-50.5	-9.8	1
MCF7	PTD - TLZ	-14.8	10.4	-35.2	5.6	1
MCF7	PTD - DAC	-37.4	10.4	-57.8	-17.0	1
MCF7	POD - Pano	-36.4	10.4	-56.8	-16.0	1
MCF7	POD - Ola	-21.6	10.4	-42.0	-1.3	1

MCF7	POD - DAC	-43.6	10.4	-64.0	-23.3	0.62
MCF7	STD - SAHA	-29.8	10.4	-50.2	-9.5	1
MCF7	STD - TLZ	-7.9	10.4	-28.3	12.4	1
MCF7	STD - DAC	-30.5	10.4	-50.9	-10.2	1
MCF7	SOD - SAHA	-39.9	10.4	-60.3	-19.5	0.88
MCF7	SOD - Ola	-18.6	10.4	-39.0	1.8	1
MCF7	SOD - DAC	-40.6	10.4	-61.0	-20.2	0.83

B. Ovarian cell lines

CellLine	Contrast	Estimate	SE	CI95Min	CI95Max	HommelPValue
HEY T30	PT - Pano	-22.6	12.9	-47.9	2.7	1
HEY T30	PT - TLZ	6.6	12.9	-18.7	31.9	1
HEY T30	PO - Pano	-1.8	12.9	-27.1	23.5	1
HEY T30	PO - Ola	-1.0	12.9	-26.3	24.3	1

HEY T30	ST - SAHA	-46.8	12.9	-72.1	-21.5	0.89
HEY T30	ST - TLZ	-10.6	12.9	-35.9	14.7	1
HEY T30	SO - SAHA	-10.6	12.9	-35.9	14.7	1
HEY T30	SO - Ola	-2.8	12.9	-28.1	22.5	1
HEY T30	PTD - Pano	-95.0	12.9	-120.3	-69.7	0.005
HEY T30	PTD - TLZ	-65.9	12.9	-91.2	-40.6	0.12
HEY T30	PTD - DAC	-74.4	12.9	-99.7	-49.1	0.046
HEY T30	POD - Pano	-49.2	12.9	-74.5	-23.9	0.65
HEY T30	POD - Ola	-48.5	12.9	-73.8	-23.2	0.72
HEY T30	POD - DAC	-28.6	12.9	-53.9	-3.3	1
HEY T30	STD - SAHA	-99.3	12.9	-124.6	-74.0	0.003
HEY T30	STD - TLZ	-63.2	12.9	-88.4	-37.9	0.17

HEY T30	STD - DAC	-71.7	12.9	-97.0	-46.4	0.063
HEY T30	SOD - SAHA	-54.1	12.9	-79.4	-28.8	0.39
HEY T30	SOD - Ola	-46.4	12.9	-71.7	-21.1	0.93
HEY T30	SOD - DAC	-26.5	12.9	-51.8	-1.2	1
SKOV-3	PT - Pano	-18.0	7.7	-33.1	-3.0	1
SKOV-3	PT - TLZ	-5.2	7.7	-20.3	9.8	1
SKOV-3	PO - Pano	-30.4	7.7	-45.5	-15.4	0.41
SKOV-3	PO - Ola	-13.9	7.7	-29.0	1.1	1
SKOV-3	ST - SAHA	-0.2	7.7	-15.3	14.8	1
SKOV-3	ST - TLZ	-3.5	7.7	-18.5	11.6	1
SKOV-3	SO - SAHA	-14.9	7.7	-30.0	0.1	1
SKOV-3	SO - Ola	-14.5	7.7	-29.5	0.6	1
SKOV-3	PTD - Pano	-85.1	7.7	-100.2	-70.0	<.0001
SKOV-3	PTD - TLZ	-72.3	7.7	-87.4	-57.2	0.0003
SKOV-3	PTD - DAC	-59.5	7.7	-74.5	-44.4	0.002
SKOV-3	POD - Pano	-90.8	7.7	-105.8	-75.7	<.0001
SKOV-3	POD - Ola	-74.3	7.7	-89.3	-59.2	0.0002

SKOV-3	POD - DAC	-65.1	7.7	-80.2	-50.1	0.0009
SKOV-3	STD - SAHA	-47.2	7.7	-62.3	-32.1	0.017
SKOV-3	STD - TLZ	-50.4	7.7	-65.5	-35.4	0.009
SKOV-3	STD - DAC	-37.6	7.7	-52.7	-22.5	0.1
SKOV-3	SOD - SAHA	-48.0	7.7	-63.0	-32.9	0.014
SKOV-3	SOD - Ola	-47.5	7.7	-62.6	-32.4	0.016
SKOV-3	SOD - DAC	-38.4	7.7	-53.4	-23.3	0.08

Supplementary Table 3. Cytotoxicity model-adjusted differences (Contrast) between drug combinations and their component drugs, listed in Figure 4A, Assay, Estimate of the contrast, Standard Error (SE) with 95% confidence intervals (CI) and Hommel-adjusted p-values. Assays were modeled independently. Separate tables for breast (A) and ovarian (B) cultures.

A. Breast cell lines

CellLine	Assay	Contrast	Estimate	SE	CI95Min	CI95Max	HommelPValue
MDAMB231	MTT	PTD - Pano	-23.6	2.3	-28.1	-19.1	<.0001
MDAMB231	MTT	PTD - TLZ	-23.8	2.4	-28.4	-19.1	<.0001
MDAMB231	MTT	PTD - DAC	-32.2	2.4	-36.8	-27.5	<.0001
MDAMB231	MTT	POD - Pano	-22.0	2.3	-26.5	-17.5	<.0001
MDAMB231	MTT	POD - Ola	-26.7	2.4	-31.3	-22.0	<.0001
MDAMB231	MTT	POD - DAC	-30.7	2.4	-35.3	-26.0	<.0001
MDAMB231	MTT	STD - SAHA	-28.6	2.3	-33.1	-24.2	<.0001
MDAMB231	MTT	STD - TLZ	-23.1	2.4	-27.8	-18.5	<.0001
MDAMB231	MTT	STD - DAC	-31.5	2.4	-36.2	-26.9	<.0001
MDAMB231	MTT	SOD - SAHA	-29.2	2.3	-33.6	-24.7	<.0001

MDAMB231	MTT	SOD - Ola	-28.1	2.4	-32.7	-23.5	<.0001
MDAMB231	MTT	SOD - DAC	-32.1	2.4	-36.7	-27.4	<.0001
MDAMB231	Ann V	PTD - Pano	30.0	3.6	23.0	37.0	<.0001
MDAMB231	Ann V	PTD - TLZ	19.3	3.8	11.8	26.8	0.0001
MDAMB231	Ann V	PTD - DAC	20.6	3.6	13.6	27.6	<.0001
MDAMB231	Ann V	POD - Pano	28.8	3.6	21.7	35.8	<.0001
MDAMB231	Ann V	POD - Ola	19.8	3.8	12.3	27.3	0.0001
MDAMB231	Ann V	POD - DAC	19.3	3.6	12.3	26.3	0.0001
MDAMB231	Ann V	STD - SAHA	31.7	3.6	24.7	38.7	<.0001
MDAMB231	Ann V	STD - TLZ	19.8	3.8	12.3	27.2	0.0001
MDAMB231	Ann V	STD - DAC	21.1	3.6	14.1	28.1	<.0001
MDAMB231	Ann V	SOD - SAHA	30.1	3.6	23.0	37.1	<.0001
MDAMB231	Ann V	SOD - Ola	19.8	3.8	12.3	27.3	0.0001
MDAMB231	Ann V	SOD - DAC	19.4	3.6	12.4	26.4	0.0001
MCF7	MTT	PTD - Pano	-18.0	4.2	-26.3	-9.7	0.003
MCF7	MTT	PTD - TLZ	-18.1	4.1	-26.1	-10.1	0.003
MCF7	MTT	PTD - DAC	-32.2	4.0	-40.0	-24.4	<.0001
MCF7	MTT	POD - Pano	-17.5	4.2	-25.8	-9.2	0.003
MCF7	MTT	POD - Ola	-19.5	4.0	-27.3	-11.7	0.0009
MCF7	MTT	POD - DAC	-31.7	4.0	-39.5	-23.9	<.0001

MCF7	MTT	STD - SAHA	-22.3	4.0	-30.1	-14.5	0.0001
MCF7	MTT	STD - TLZ	-19.6	4.1	-27.6	-11.6	0.001
MCF7	MTT	STD - DAC	-33.6	4.0	-41.5	-25.8	<.0001
MCF7	MTT	SOD - SAHA	-22.7	4.0	-30.5	-14.8	<.0001
MCF7	MTT	SOD - Ola	-21.8	4.0	-29.6	-14.0	0.0002
MCF7	MTT	SOD - DAC	-34.0	4.0	-41.8	-26.2	<.0001
MCF7	Ann V	PTD - Pano	21.0	4.7	11.8	30.2	0.011
MCF7	Ann V	PTD - TLZ	16.7	4.7	7.6	25.9	0.068
MCF7	Ann V	PTD - DAC	20.7	4.7	11.5	29.8	0.014
MCF7	Ann V	POD - Pano	19.8	4.7	10.7	29.0	0.021
MCF7	Ann V	POD - Ola	15.4	4.7	6.2	24.6	0.1
MCF7	Ann V	POD - DAC	19.5	4.7	10.3	28.7	0.022
MCF7	Ann V	STD - SAHA	22.8	4.7	13.7	32.0	0.004
MCF7	Ann V	STD - TLZ	18.4	4.7	9.3	27.6	0.031
MCF7	Ann V	STD - DAC	22.4	4.7	13.2	31.5	0.005
MCF7	Ann V	SOD - SAHA	18.2	4.7	9.0	27.4	0.036
MCF7	Ann V	SOD - Ola	13.6	4.7	4.4	22.8	0.13
MCF7	Ann V	SOD - DAC	17.7	4.7	8.5	26.9	0.046

B. Ovarian cell lines

CellLine	Assay	Contrast	Estimate	SE	CI95Min	CI95Max	HommelPValue
HEY T30	MTT	PTD - Pano	-34.4	4.4	-43.0	-25.7	<.0001
HEY T30	MTT	PTD - TLZ	-49.4	4.3	-57.8	-40.9	<.0001
HEY T30	MTT	PTD - DAC	-35.5	4.3	-43.9	-27.1	<.0001
HEY T30	MTT	POD - Pano	-30.3	4.4	-39.0	-21.7	<.0001
HEY T30	MTT	POD - Ola	-44.5	4.3	-52.9	-36.1	<.0001
HEY T30	MTT	POD - DAC	-31.5	4.3	-39.9	-23.1	<.0001
HEY T30	MTT	STD - SAHA	-41.6	4.3	-50.0	-33.2	<.0001
HEY T30	MTT	STD - TLZ	-48.0	4.3	-56.4	-39.6	<.0001
HEY T30	MTT	STD - DAC	-34.1	4.3	-42.6	-25.7	<.0001
HEY T30	MTT	SOD - SAHA	-42.3	4.3	-50.7	-33.9	<.0001
HEY T30	MTT	SOD - Ola	-47.9	4.3	-56.3	-39.4	<.0001
HEY T30	MTT	SOD - DAC	-34.8	4.3	-43.3	-26.4	<.0001
HEY T30	Ann V	PTD - Pano	29.9	6.0	18.1	41.7	0.0008
HEY T30	Ann V	PTD - TLZ	32.6	6.0	20.8	44.4	0.0003
HEY T30	Ann V	PTD - DAC	29.3	6.0	17.4	41.1	0.001
HEY T30	Ann V	POD - Pano	23.6	6.0	11.8	35.4	0.01
HEY T30	Ann V	POD - Ola	30.6	6.0	18.8	42.4	0.0006
HEY T30	Ann V	POD - DAC	22.9	6.0	11.1	34.8	0.01
HEY T30	Ann V	STD - SAHA	33.1	6.0	21.3	44.9	0.0002
HEY T30	Ann V	STD - TLZ	32.6	6.0	20.7	44.4	0.0003
HEY T30	Ann V	STD - DAC	29.2	6.0	17.4	41.0	0.001
HEY T30	Ann V	SOD - SAHA	28.0	6.2	15.8	40.2	0.004
HEY T30	Ann V	SOD - Ola	31.8	6.2	19.6	43.9	0.0006
HEY T30	Ann V	SOD - DAC	24.1	6.2	11.9	36.3	0.01

SKOV-3	MTT	PTD - Pano	-22.5	4.4	-31.1	-13.8	0.0004
SKOV-3	MTT	PTD - TLZ	-26.6	4.4	-35.3	-18.0	<.0001
SKOV-3	MTT	PTD - DAC	-44.0	4.4	-52.7	-35.4	<.0001
SKOV-3	MTT	POD - Pano	-20.1	4.4	-28.7	-11.4	0.0008
SKOV-3	MTT	POD - Ola	-27.2	4.4	-35.8	-18.5	<.0001
SKOV-3	MTT	POD - DAC	-41.7	4.4	-50.3	-33.0	<.0001
SKOV-3	MTT	STD - SAHA	-21.4	4.3	-29.8	-13.1	0.0005
SKOV-3	MTT	STD - TLZ	-22.0	4.4	-30.7	-13.4	0.0005
SKOV-3	MTT	STD - DAC	-39.4	4.4	-48.1	-30.8	<.0001
SKOV-3	MTT	SOD - SAHA	-19.7	4.3	-28.1	-11.3	0.0008
SKOV-3	MTT	SOD - Ola	-23.2	4.4	-31.9	-14.6	0.0003
SKOV-3	MTT	SOD - DAC	-37.7	4.4	-46.4	-29.1	<.0001
SKOV-3	Ann V	PTD - Pano	18.2	5.1	8.3	28.2	0.037
SKOV-3	Ann V	PTD - TLZ	24.6	4.9	15.0	34.1	0.001
SKOV-3	Ann V	PTD - DAC	32.2	4.9	22.6	41.7	<.0001
SKOV-3	Ann V	POD - Pano	17.3	5.1	7.3	27.2	0.037
SKOV-3	Ann V	POD - Ola	24.4	5.1	14.4	34.3	0.002
SKOV-3	Ann V	POD - DAC	31.2	4.9	21.7	40.8	<.0001
SKOV-3	Ann V	STD - SAHA	23.7	4.9	14.2	33.3	0.002
SKOV-3	Ann V	STD - TLZ	22.9	4.9	13.3	32.4	0.003
SKOV-3	Ann V	STD - DAC	30.5	4.9	20.9	40.0	<.0001
SKOV-3	Ann V	SOD - SAHA	22.6	5.1	12.7	32.6	0.006
SKOV-3	Ann V	SOD - Ola	22.5	5.3	12.2	32.8	0.008
SKOV-3	Ann V	SOD - DAC	29.3	5.1	19.4	39.3	0.0001

Supplementary Table 4. List of primary antibodies, their sources, and dilutions

Antigen	Company/Cat. #	Source*	Dilution**
β-ACTIN	Sigma/A5316	Mouse	6000
Ac H3 K9	Active Motif/39917	Rabbit	2000
Ac α-Tubulin K40	Cell Signaling/5335	Rabbit	3000
Artemis	Cell Signaling/13381	Rabbit	3000
P-ATM (S1981)	Cell Signaling/5883	Rabiit	2000
ATM	Cell Signlaing/2873	Rabbit	2500
ATRX	Cell Signlaing/10321	Rabbit	3000
BRCA1	Cell Signlaing/14823	Rabbit	2000
CHD3	Cell Signlaing/4241	Rabbit	2000
CHD4	Cell Signlaing/12011	Rabbit	2000
Cleaved CASPASE 3	Cell Signaling/9661	Rabbit	2500
Cleaved PARP1 (Asp214)	Cell Signlaing/5625	Rabbit	2000
DNA Ligase 1	GeneTex/GTX70141	Mouse	2500
P-DNAPKcs (S2056)	Cell Signaling/68716	Rabbit	2000
DNAPKcs	Cell Signlaing/4602	Rabbit	3000
HDAC1	Cell Signlaing/34589	Rabbit	2500
γ-H2AX	Cell Signlaing/2577	Rabbit	1500
MBD3	Cell Signlaing/99169	Rabbit	2000
MTA1	Cell Signlaing/5647	Rabbit	2000
c-MYC	Cell Signaling/5605	Rabbit	3000
PAR	Bio-Techne/4335-MC-100	Mouse	2000
*Used anti-rabbit or anti-mouse IgG for secondary antibody from Bio-Rad Lab.<br>**Fold dilution in PBS with 0.1% Tween 20			
